# Factors influencing the use of modern contraception among reproductive aged women in Bangka Belitung Province, Indonesia

**DOI:** 10.11604/pamj.2021.39.39.28870

**Published:** 2021-05-13

**Authors:** Antarini Antarini

**Affiliations:** 1 Department of Midwifery, Health Polytechnic Pangkalpinang, Bangka Belitung, Indonesia

**Keywords:** Contraception, determinant, family planning, knowledge, women

## Abstract

**Introduction:**

contraceptives in family planning are used to control the timings between pregnancies. Although the number of those using family planning has increased, determinants of contraceptive use among women in Indonesia remain insufficient. This research aimed to identify the factors associated with contraceptive use among reproductive aged women in Bangka Belitung Province.

**Methods:**

this study employed data from the Indonesian demographic and health survey (IDHS) 2017. The selected respondents were 768 women aged 15-49 years. Then, the determinants of contraceptive use among women were examined by multinomial logistic regression.

**Results:**

women’s aged 15-49 years (adjusted Odds Ratio (aOR) =8.955; 95% CI=3.573-22.439), level of education (aOR=2.017; 95% CI=1.053-3.862), the number of children (aOR=1.207; 95% CI=0.498-2.926), residential location (aOR=0.877; 95% CI=0.601-1.282), wealth index (aOR=2.23; 95% CI=0.953-5.218), visited health facilities (aOR=1.683; 95% CI=1.174-2.412), knowledge of contraceptive method (aOR=2.043; 95% CI=2.043-2.043) were significantly associated with contraceptive use among reproductive age women.

**Conclusion:**

factors such as women’s age, education, number of living children, area of residence, wealth index, knowledge, and visits to health facilities were still considered significant issues in determining contraceptive use among reproductive-age women in Bangka Belitung Province.

## Introduction

According to the world population data sheet 2013, Indonesia is the 5^th^ country in the world with the highest projected population, which is 249 million. In 2017, the Indonesian demographic health survey (IDHS) showed a total fertility rate (TFR) of 2.4 children per woman. This number is the same as the average TFR of the Asian countries. It indicates that Indonesian women give birth to an average of 2.4 children during her lifetime if she follows the current age specific fertility rate (ASFR) trend. In rural areas, the overall fertility rate is even higher, i.e. 2.6 million. It is 13 percent higher than those who live in urban areas (2.3 children) [1]. Faced with this fact, high population growth is the main problem in Indonesia. When the population growth rate gets higher, the effort made to maintain the people's welfare is also greater. Hence, the Indonesian government continues to strive to reduce the growth rate through the family planning program. The high population growth will hamper the pace of development in various fields. Therefore, efforts are needed to reduce the birth rate. One of the ways to overcome this high population growth rate is the family planning program [2]. The Indonesian government has committed to balancing the nation´s population through the family planning program [2]. The success of the family planning program in Indonesia is determined by several factors, including effectiveness, safety, frequency of use, side effects, and the willingness and ability to perform contraception regularly and correctly. Apart from that, consideration of contraception is also based on the cost and the role of religion and culture regarding contraception. To achieve the goal, the government has tried to socialize the family planning program. However, many fertile aged couples are still reported not to use contraception, even though they need contraception, so called unmet need [3]. In accordance with the normative definition of the demographic and health survey (DHS), the family planning group with the unmet need is all pregnant women who are married or living together, sexually active partners, do not want children or births at least two years without the use of contraceptives. Based on the IDHS in 2017, the number of unmet needs in Indonesia reached 11.4 percent with 4.5 percent for spacing and 6.9 percent for spacing. Compared to the findings of the previous survey, this figure increased to 8.6 percent in 2007. The prevalence of contraceptive use increased from 50 percent in 1991 to 64 percent in 2017. Nevertheless, in the last 10 years, the use of modern contraception experiences a slower increase by only 2 percent [1].

In Indonesia, the proportion of women in need of family planning services is around 73 percent; 85 percent of them are happy. When all the needs for family planning (FP) services are met, the contraception prevalence can be increased from 62 percent to 73 percent. As many as 88 percent of currently married women have the same criteria for FP facilities as in 2007 (87%). It means that the number of new accommodations is just one percent. In other words, the number of new acceptors has not risen significantly. The reasons for the high unmet need are not only caused by socio-demographic and economic conditions but also due to access to services, quality of family planning supplies and services, lack of information, family and community tensions, spouse, family and community barriers, and low expectations of pregnancy risks [1]. Nationally, the use of contraception among women aged 15-19 and 45-49 years old is lower than those aged 20-44 years old. One of the reasons is the fact that young women tend to use modern, short-term contraceptives such as injections and birth control pills. Meanwhile, those who are older tend to use long-term contraceptives such as intrauterine device (IUDs) and female sterilizers. Nowadays, the trend of modern contraceptive methods increases, especially among female prostitute, from 47% in the 1991 IDHS to 57% in the 2017 IDHS. However, the percentage of episodes of contraceptive methods that were stopped within 12 months (dropout rate) in the 2007-2017 IDHS has also increased in the modern contraceptives, namely pill and injection [1]. In 2017, the IDHS reported that people who used contraceptives in Indonesia were 63.6% and those who did not use contraception were 36.4%. When we look closer at the level of the provincial distribution, the survey showed that the highest percentage of the married couple who used contraceptives was in North Maluku province (87.03%), followed by the provinces of Bangka Belitung (83.92%) and North Sulawesi (83.84%) [1]. In Bangka Belitung, 62.2% of married women using modern contraceptives and 8.9% used other contraceptives. The contraceptive prevalence rate (CPR) reached 81.6% and the unmet need in Bangka Belitung in 2018 was 8.31%. Also, it was reported that women in Bangka Belitung control the birth through various means such as injection 32.1%, tubectomy 3.8%, vasectomy 0.1%, 4.7% implant/contraceptive implant, pill 15.7%, spiral 3.3%, condom 2,4%, and lactational amenorrhea method (LAM) method 0.1%. Therefore, the researchers aim to conduct research using secondary data from the 2017 IDHS. The present study examines the factors of contraceptive uses among reproductive-age women in Bangka Belitung Province.

## Methods

This research employed data that was collected from 2017 by the Indonesia demographic and health survey (IDHS). The survey was conducted by the Central Statistics Agency in collaboration with the National Population and Family Planning Agency and the Ministry of Health. Technical assistance was provided by the inner city fund (ICF) through the demographic project and health surveys (DHS) Program. The present research determined certain criteria to select participants as the sample, such as married women aged 15-49 years who were using contraception in Indonesia. In addition, the participants must complete and return the questionnaires properly. The survey successfully interviewed 768 women as the initial sample in Bangka Belitung Province. They were also requested to answer a questionnaire for this research purpose. Based on the inclusion criteria, 375 women remained. The IDHS used two-stage stratified cluster sampling to select the sample, including 1970 census blocks covering urban and rural areas. This research had dependent and explanatory variables. The dependent variable included contraceptive uses, which was defined as the use of contraception by women aged 15-49 years by the time the survey was carried out. For the explanatory variables, we used several factors like age, the number of living children, educational level, occupation, wealth index, residential location, access to the information, knowledge toward contraceptive methods, visits health facilities, and visits by fieldworker. Having collected the data, they were then analyzed using the SPSS statistical software version 22. Before the analysis began, the dataset was weighted to account for any differences, considering the nature of the sampling design method. Univariate and bivariate analysis was done using the chi-square test. Meanwhile, the multinomial logistic regression analysis was carried out to examine the determinants of contraceptive use among women aged 15-49 years.

## Results

This research reported interesting results since most women aged 15-49 years, (69.5%) both modern and traditional methods, used a family planning (FP) technique. The users of the modern family planning technique were women of reproductive age, ranging from the age of 15-19 years old (2%) to 30-34 years old (24%). However, the rate fell to 12% in participants aged 45-49 years. The highest educational background of women surveyed in this research was the high school level. Meanwhile, the lowest educational level of those who used contraceptive in this research was women who did not attend school. The number was quite high by 164 individuals (48%). Regarding occupation, more than half of the reproductive-age women were reported to work (55%). In this context, working women were more likely to use contraceptives than those who did not work. Further, reproductive-age women with less than 2 born-alive children were more likely to use modern family planning methods (95%) compared to those who had more than 2 born-alive children. The research also revealed that women of reproductive age who lived in urban areas (57%) were relatively wealthier than women living in rural areas (57%). The highest wealth index was found in women who were grouped in the middle and upper-middle-income quintiles. In other words, the low wealth quintile of the reproductive age women might lead to a lower use of contraceptive modern methods. Furthermore, this research found that television was the most widely accessed sources that participants used to gather information about family planning. In contrast, radio, magazine, and newspapers were less accessible to women. The leading advertisements, entitled let's join family planning, was broadcasted on television intensively. Thus, the family planning information could be easily obtained by the public. Meanwhile, information that was delivered through radio, newspapers, magazines have begun to be abandoned.

The present research also investigated the frequency of visits to health facilities. The research found that some women who used the family planning technique did not visit health facilities (43%). The majority of women who used family planning techniques or methods attended family planning field officers and health centre. Regarding the contraceptive methods, pills (96%) were documented as the most popular methods among women who used the family planning methods. The knowledge of birth spacing and Family Planning is an important aspect to better understand the variety of contraceptives. Furthermore, this knowledge will affect the use of appropriate and effective contraceptives. In this research, participants' knowledge about contraceptive methods was obtained by asking them all types of contraceptives that they have ever heard to delay or avoid pregnancy and birth. For assessing the effectiveness of the family planning program, information on the use of contraceptives is significant. This information was collected by asking whether a contraceptive device or procedure was used by the respondent or his partner during the interview. Data are presented in Table 1. In the bivariate analysis, all of the variables, except for occupation, information sources and visitation, were significantly associated with contraceptive use among married women (Table 2).

**Table 1:** socio-demographic characteristics of the Bangka Belitung women

Variable	n	%
**Contraception**		
Use	429	55.86
Not use	339	44.14
**Age**		
15-19	138	17.97
20-24	97	12.63
25-29	100	13.02
30-34	129	16.80
35-39	105	13.67
40-44	113	14.71
45-49	86	11.20
**Education**		
Higher	108	14.06
Secondary	425	55.34
Primary	222	28.91
No education	13	1.69
**Occupation**		
No	353	45.96
Yes	415	54.04
**Number of living children**		
0-2	573	74.61
3-4	164	21.35
5+	31	4.04
**Residential location**		
Urban	458	59.64
Rural	310	40.36
**Wealth Index**		
Poorest	45	5.86
Poorer	136	17.71
Middle	174	22.66
Richer	206	26.82
Richest	207	26.95
**Access to the information**		
No	271	35.29
Yes	497	64.71
**Visit to health facility**		
No	293	38.15
Yes	103	13.41
**Visited by fieldworker**		
No	13	1.69
Yes	34	4.43
**Knowledge of contraception**		
Not know	10	1.30
Traditional method	1	0.13
Modern method	757	98.57
		

**Table 2:** bivariate analysis of the determinants of contraceptive use among women in Bangka Belitung Province

Variable	Contraception				X2
	Use		Not use		
	n	%	n	%	
**Age**					
15-19	8	2.36	130	0.30	192.391*
20-24	28	8.26	69	16.08	
25-29	51	15.04	49	11.42	
30-34	82	24.19	47	0.96	
35-39	59	17.40	46	10.72	
40-44	70	20.65	43	10.02	
45-49	41	12.09	45	10.49	
**Education**					
Higher	33	9.73	75	17.48	45.723*
Secondary	164	48.38	261	60.84	
Primary	138	40.71	84	19.58	
No education	4	1.18	9	2.10	
**Occupation**					
No	151	44.54	202	47.09	123.00
Yes	188	55.46	227	52.91	
**Number of living children**					
0-2	208	61.36	365	5.08	67.508*
3-4	114	33.63	50	11.66	
5+	17	5.01	14	3.26	
**Residential location**					
Urban	192	56.64	266	62.00	10.238*
Rural	147	43.36	163	38.00	
**Wealth index**					
Poorest	23	6.78	22	5.13	12.036*
Poorer	69	20.35	67	15.62	
Middle	87	25.66	87	20.28	
Richer	89	26.25	117	7.27	
Richest	71	20.94	136	31.70	
**Access to the information**					
No	110	32.45	161	37.53	9.7710
Yes	229	67.55	268	62.47	
**Knowledge of contraception**					
Not know	0	-	10	2.33	23.832*
Traditional method	0	-	1	0.23	
Modern Method	339	100.00	418	97.44	
**Visit to health facility**					
No	148	145	293	8.15	9.771*
Yes	36	67	103	3.41	
**Visited by fieldworker**					
No	4	9	13	1.69	3.3020
Yes	17	17	34	0.43	


* p-value<0.05; **p-value<0.01: ***p-value<0.001

In the multivariate analysis, the association between the independent and dependent variables was assessed by multinomial logistic regression (Table 3). This research revealed that women aged 15-19 years were more likely to use contraceptives than women aged 45-49 years (adjusted Odds Ratio (aOR) =8.955; 95% CI=3.573-22.439). Additionally, women who had completed higher education had 2 times greater odds of using contraceptives (aOR=2.017; 95% CI=1.053-3.862) than those who only had a primary school background and had not completed any formal education at all. Following that, women who had 1-2 children were more likely to use contraception than those who had more than 5 children (aOR=1.207; 95% CI=0.498–2.926). Then, Women who lived in cities would more probably use contraceptives than women who lived in a rural area (aOR=0.877; 95% CI=0.601-1.282). Regarding wealth, women that were classified the richest by the wealth index were projected 2.23 times higher to use contraceptives than those classified as the poorest (aOR=2.23; 95% CI=0.953–5.218). After that, women who did not visit health facilities would be more likely to use contraceptives (aOR=1.683; 95% CI=1.174-2.412) than those who visited health facilities. Finally, the researchers reported that women who have sufficient knowledge about traditional methods were more likely to use contraceptives than those who did not know any method (aOR=2.043; 95% CI=2.043-2.043)

**Table 3:** multinomial logistic regression of the determinants of contraceptive use among women in Bangka Belitung Province

Variable	X2	aOR	95%CI	
			Lower	Upper
**Age**				
15-19	192.391*	8.955	3.573	22.439
20-24		1.634	0.789	3.384
25-29		0.558	0.279	1.117
30-34		0.307	0.158	0.6
35-39		0.522	0.269	1.013
40-44		0.428		
45-49		-	0.224	0.817
**Education**				
Higher	45.723*	2.017	1.053	3.862
Secondary		1.283		
Primary		-		
No education		-	0.834	1.973
**Number of living children**				
1-2	67.508*	1.207	0.498	2.926
3-4		0.496		
5+		-	0.201	1.219
**Place of residence**				
Urban	10.238*	0.877		
Rural		-	0.601	1.282
**Wealth index**				
Poorest	12.036*	-		
Poorer		1.019	0.445	2.337
Middle		1.317	0.584	2.97
Richer		1.534	0.68	3.46
Richest		2.23	0.953	5.218
**Visited health facilities**				
No	9.771*	1.683		
Yes		-	1.174	2.412
**Knowledge of contraception**				
Not know	23.832*	-		
Traditional method		2.043	2.043	2.043
Modern method				
*p-value<0.05; **p-value<0.01: ***p-value<0.001		-		

## Discussion

Contraception methods or procedures are classified into two groups, namely modern and traditional methods. Female sterilization, male sterilization, birth control pills, intrauterine device (IUD), contraceptive injections, implants, male contraceptives, intravag, diaphragm, emergency contraception, and lactation amenorrhea method (LAM) approaches belong to the former method. Meanwhile, periodic abstinence or calendar, interrupted sex, and herbal medicine represents the latter. One of the provisions that women should into account when choosing contraception is valid information. Contraceptive pills and injection are the most familiar contraceptive methods by the respondents with almost the same percentage i.e. 99%. The respondent's knowledge of traditional contraceptive methods of periodic abstinence was better known by the respondents with a percentage of 42% followed by discontinuation of 37%. This shows that if the data was obtained easily, the information would be better. On the other hand, when the awareness was lesser, the growth of an individual will be hindered by the values implemented. Health information is very important for all of us. One way to obtain information is through the internet, books, health magazines, or health workers who often provide counseling. The benefits of using contraceptives have been felt by the community to create a happy and prosperous small family. Thus, there seemed an increasing number of family who had 1-2 children, especially among women who worked to help their husbands earn money. It also shows the progress made by the Indonesian government in implementing the FP program. This success has been widely recognized and considered as one of the "centers of excellence" in the field of population. Results of this research also showed that age was significantly associated with the use of contraceptives among reproductive-age women. This result is consistent with studies conducted in Ghana and Nigeria. The studies documented that older women had a lower level of concern with modern contraceptive use [4,5] It could be caused by their lower fecundity rates and less active sexual desires [6]. In fact, using contraception is beneficial to delay or give space for subsequent pregnancies and to limit the number of children [7,8,9].

Educational level was significantly associated with contraceptive use among these reproductive-age women. For instance, women with higher education degrees were correlated with higher use of contraceptives. Two previous studies conducted in Bangladesh and Ghana reported that education had an extremely significant influence on contraceptive use. In this report, women with higher education were more likely to use contraception than those without formal education [10,11]. This finding emphasized that highly educated people were probably more aware of the benefits and importance of using contraception [10]. Another study in Nigeria also found that educated women were more likely to use contraceptives [5]. Likewise, Misnaniarti and Ayuningtyas (2016) and Sharjabad *et al*. (2013) agreed that a person's education level affected contraceptive use [12,13]. If the educational level was low, the use of contraception was also low. In short, the level of education affects a person in using the contraceptive method. Women of reproductive age with higher education will use the long-term contraceptive method compared to those with low education. It is evidenced by research from Allen, Cwiak, and Kaunitz (2013) stating that women with higher education degrees used modern contraceptives [14]. Besides the educational background, the use of contraceptives was also associated with the number of children. For instance, women with five or more living children were more likely to use contraceptives. This finding is consistent with studies conducted in Ghana [4,15]. The addition of one child will increase the tendency of married women to use contraception by 7%-8%. Another study also found that women with more than three living children were more likely to use contraceptives than those without children. One of the reasons for using contraceptives was to prevent them from having more children [5,6,10,11].

Women will choose to use contraception when they have reached their ideal family size [4]. Therefore, women who have many children have more chances to use contraceptives because they have reached the ideal size of the family. According to Amran and Damayanti (2018), women who have two or more living children had a bigger desire to limit births. As a result, they were triggered to use contraception [4]. In this research, there was a strong relationship between the number of children and the use of FP. Another interesting result was also reported regarding the place where the women were living. It showed a significant relationship with contraceptive use. When deciding to use contraceptives, spouses of reproductive age was likely influenced by the area where they are living. Women who reside in urban areas tend to have easier access to health facilities. Additionally, the information about contraceptive methods will be easier to access. Meanwhile, in rural areas, women have limited coverage of health facilities because very few health facilities are located in rural areas. Women of reproductive age who live in rural areas use more hormonal contraceptives than those in urban areas. The possible reason is that they want practical contraceptives that do not make them repeatedly come to health service facilities to obtain contraceptive services. In addition, contraception is very effective in preventing pregnancy. Women from the richest tier of the wealth index also had odds that progressively increased to using a contraceptive. Similar findings regarding the strong association between wealth index and contraceptive use were obtained by studies conducted in Malawi and Ghana. In these countries, women classified within the richest wealth index were more likely to use contraception than those grouped in the poorest index [4,5,11-16]. In 2017, Ofonime claimed that financial factors played an important role in the decreased use of contraceptives among the poorest married women [17]. Providing free access for contraceptives to poor women would be beneficial to increase the use of contraceptives. Besides wealth, knowledge of contraception was also found to be statistically significant. There was a correlation between knowledge of family planning techniques and the use of contraceptives among reproductive-age women. Knowledge is believed as one of the factors that influence the decision to use contraception.

However, occupation showed no association between contraceptive use among these married women. Economically, working women will benefit from enhancing the family economy and at the same time improving health financing, including support for interest-based hormonal contraceptives. Women's occupation has an impact on fertility and contraceptive use. Thus, contraception for working women is very useful for regulating and limiting births. It can then lead to support the women´s work careers, especially for women who work outside the home as paid employees and tend to have fewer children than those who do not work. Meanwhile, access to information showed no association with contraceptive use among these married women. Mubashar *et al*.in 2016, for example, found that most sources of contraception information were from media, such as television, radio, the Internet, local news, newspaper, and magazine [18]. Besides, access to information from television was significantly associated with contraceptive use. Women who watched television had approximately 1.5 greater odds of using contraceptives than those who never watch television. This finding is consistent with the studies conducted in Ethiopia and Ghana [19,20,21]. Exposure to media, such as television, is an important factor that affects women´s knowledge about contraceptive use [19]. As mentioned earlier, watching television is important to increase the knowledge of the wider community to understand the types, benefits, and methods of using contraception correctly. In contrast, this study showed that access to information via the Internet was significantly associated with less likelihood of contraceptive use among married women. This finding is congruent with a study conducted in Australia [22]. In the study, 50% of women reported that they felt dissatisfied with the quality and quantity of information about contraception that they obtained from the Internet. The best approach to encourage contraceptive use and avoid bias information from unreliable media sources could be done by improving the sources of information about contraceptives.

## Conclusion

The rate of women in Bangka Belitung who actually used modern family planning was 44.1%. Most women who used family planning were 30-34 years and have graduated from high school. They also had careers, 1-2 children, and lived in urban and rural areas. The 3-month family planning injection was the main contraceptive used in Bangka Belitung Province by women of reproductive age, accompanied by birth control pills. Factors such as women´s age, education, number of living children, area of residence, wealth index, knowledge, and visits to health facilities were still considered significant issues in determining contraceptive use among reproductive-age women in Bangka Belitung Province. Overall, the study results suggest that policymakers should target certain women and create a campaign regarding contraception. Targeting older, poor, and uneducated or less educated women may have a positive impact in terms of increasing their use of contraception.

### What is known about this topic

- The pills, were documented as the most popular methods among women who used the Family Planning methods;
- The knowledge about contraceptive methods was obtained by asking them all types of contraceptives that they have ever heard to delay or avoid pregnancy and birth.

### What this study adds

- The lowest educational level of those who used contraceptive was women who did not attend school;
- Working women were more likely to use contraceptives than those who did not work;
- The low wealth quintile of the reproductive age women might lead to a lower use of contraceptive modern methods.
